# Concurrent evolution of resistance and tolerance to potato virus Y in *Capsicum annuum* revealed by genome‐wide association

**DOI:** 10.1111/mpp.13157

**Published:** 2021-11-02

**Authors:** Lucie Tamisier, Marion Szadkowski, Grégory Girardot, Caroline Djian‐Caporalino, Alain Palloix, Judith Hirsch, Benoit Moury

**Affiliations:** ^1^ Pathologie Végétale INRAE Montfavet France; ^2^ GAFL INRAE Montfavet France; ^3^ Institut Sophia Agrobiotech INRAE Université Côte d'Azur CNRS Sophia Antipolis France

**Keywords:** genome‐wide association, pepper, *Potato virus Y*, resistance, tolerance, trade‐off

## Abstract

We performed a genome‐wide association study of pepper (*Capsicum annuum*) tolerance to potato virus Y (PVY). For 254 pepper accessions, we estimated the tolerance to PVY as the coefficient of regression of the fresh weight (or height) of PVY‐infected and mock‐inoculated plants against within‐plant virus load. Small (strongly negative) coefficients of regression indicate low tolerance because plant biomass or growth decreases sharply as virus load increases. The tolerance level varied largely, with some pepper accessions showing no symptoms or fairly mild mosaics, whereas about half (48%) of the accessions showed necrotic symptoms. We found two adjacent single‐nucleotide polymorphisms (SNPs) at one extremity of chromosome 9 that were significantly associated with tolerance to PVY. Similarly, in three biparental pepper progenies, we showed that the induction of necrosis on PVY systemic infection segregated as a monogenic trait determined by a locus on chromosome 9. Our results also demonstrate the existence of a negative correlation between resistance and tolerance among the cultivated pepper accessions at both the phenotypic and genetic levels. By comparing the distributions of the tolerance‐associated SNP alleles and previously identified PVY resistance‐associated SNP alleles, we showed that cultivated pepper accessions possess favourable alleles for both resistance and tolerance less frequently than expected under random associations, while the minority of wild pepper accessions frequently combined resistance and tolerance alleles. This divergent evolution of PVY resistance and tolerance could be related to pepper domestication or farmer's selection.

## Graphical abstract

Among a core‐collection of pepper accessions, phenotypic and genetic negative correlations were found between resistance and tolerance to PVY for the cultivated pepper accessions, but not for the wild pepper accessions.

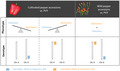

## 1 INTRODUCTION

To avoid or mitigate the harmful effects of pests and pathogens, plants have developed two main lines of defence: resistance and tolerance (Råberg et al., 2007). Resistance is characterized by a reduction of parasite load within (or on) plants. In contrast, tolerance acts by reducing the plant damage caused by parasite infection, regardless of the parasite load. Most efforts in plant genetics or evolution studies have been devoted to understanding and exploiting resistance mechanisms, with major‐effect resistance genes being the key targets for plant breeders to control the negative effects of plant parasites. At the agronomic level, very few tolerance genes with a major effect have been identified by genetic studies, used on purpose in breeding programmes and deployed in the field for crop production. One notable exception is the *Zym* tolerance gene from *Cucurbita moschata*, introgressed into cultivars of zucchini (*Cucurbita pepo*). This gene confers a very strong tolerance to zuccini yellow mosaic virus (ZYMV; species *Zucchini yellow mosaic virus*; genus *Potyvirus*, family *Potyviridae*). Indeed, although the virus load is similar within zucchini plants carrying or not carrying the *Zym* gene, only the latter type of plants shows viral symptoms and a decrease of fruit production and quality compared to healthy plants (Desbiez et al., 2003).

In most situations, however, the tolerance of plants to parasites is only partial, that is, the health (or fitness, or yield in the case of crop plants) of parasite‐infected tolerant plants is intermediate between those of healthy plants and of nontolerant plants infected with parasites. In addition, plant genotypes and cultivars may carry different sets of resistance and tolerance genes and/or quantitative trait loci (QTLs). Accordingly, a quantitative measure of tolerance that allows it to be dissociated from resistance is required. The tolerance of a plant genotype to a given pathogen can be estimated by the slope of regression of plant health (or fitness) against the pathogen load within the plant. This slope is often negative, as the health of plants generally decreases as the pest load increases. Therefore, the steeper the slope, the lower the tolerance. In addition, there are cases where the slope is positive, as shown for potato virus Y (PVY; species *Potato virus Y*, genus *Potyvirus*) in some pepper genotypes (Montarry et al., 2012), a phenomenon called overcompensation (Ramula et al., 2019). This also highlights the great potential of tolerance to control plant diseases in an agricultural context, tolerant genotypes being able to maintain their health (or fitness) level whatever the pathogen load.

Several studies have questioned the concurrent evolution of resistance and tolerance. Fineblum and Rausher (1995) were the first to examine the evolution of both traits and provided evidence for a trade‐off between resistance and tolerance to herbivory in *Ipomea purpurea*. Indeed, if plant resources are limited and if resistance or tolerance is costly, plants may be able to invest in either resistance or tolerance mechanisms, but not both. Therefore, a negative correlation between tolerance and resistance is expected. To date, numerous mathematical models have been developed and yielded insights into the evolution of these defence strategies (Best et al., 2008; Miller et al., 2005; Restif & Koella, 2004; Roy & Kirchner, 2000). However, little empirical evidence of this trade‐off has been found (Fineblum & Rausher, 1995; Mauricio et al., 1997; Stowe, 1998), and only one phenotypic trade‐off has been demonstrated for a plant pathogen (Mikaberidze & McDonald, 2020).

As there is a general need to better understand the mechanisms of tolerance, its role in the (co)evolution of plants and pathogens and its relevance to plant breeding (Pagán & García‐Arenal, 2020), we carried out a genetic analysis of tolerance to PVY in a core‐collection of 276 genotyped accessions representative of pepper (*Capsicum annuum*, family Solanaceae) diversity. PVY is an RNA virus that is transmitted by aphid vectors in the field and induces serious diseases in solanaceous crops (pepper, potato, tobacco, tomato) worldwide (Quenouille et al., 2014). We aimed to (a) examine if tolerance is a heritable trait within the pepper core‐collection, (b) study the genetic architecture underlying tolerance, (c) compare these results to PVY‐resistance QTLs previously identified in the same core‐collection of peppers (Tamisier et al., 2020), and (d) examine the joint evolution of tolerance and resistance to test the hypothesis of a trade‐off between these traits.

## 2 RESULTS

### 2.1 Variation in PVY tolerance among the pepper core‐collection

To assess tolerance to PVY among the core‐collection, measures of plant health (plant height and weight) and virus accumulation were performed for each accession. Therefore, two measurements of tolerance were obtained per accession, using either plant height or plant weight as a proxy of plant health.

**FIGURE 1:**
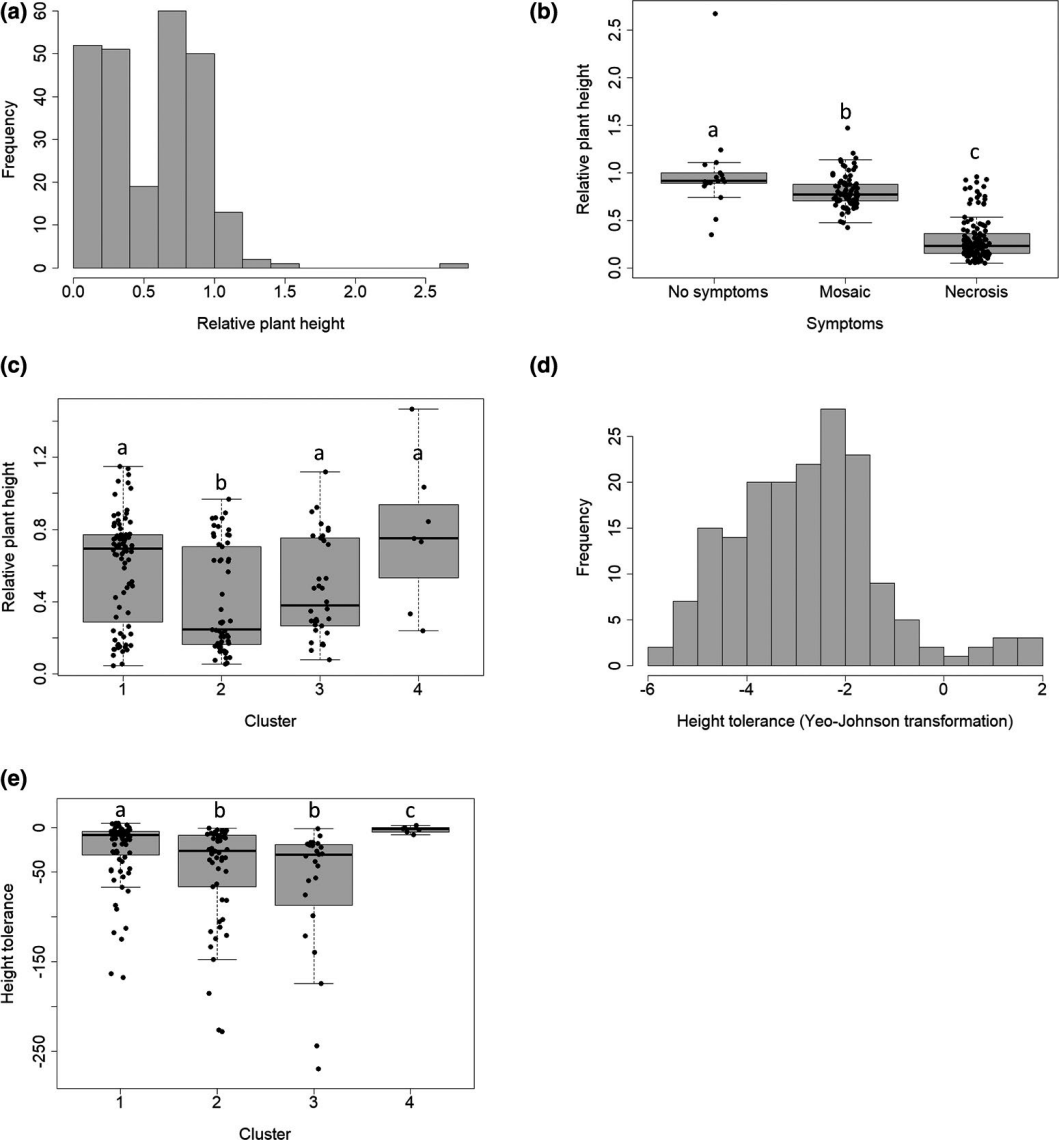
Height tolerance in the pepper core‐collection 1 month after potato virus Y inoculation. (a) Frequency distribution of the mean plant height of infected plants relative to mock‐inoculated controls of the same accession. (b) Distribution of the mean relative plant height of pepper accessions according to the systemic symptoms. (c) Distribution of the relative plant height among the four clusters of the core‐collection. (d) Frequency distribution of the height tolerance for each accession. (e) Distribution of the height tolerance among the four clusters of the core‐collection. The letters a, b, and c indicate the different groups obtained after pairwise comparisons using the Nemenyi test (*p* < 0.05)

Data on plant height and weight could be obtained for 254 of the 276 accessions of the core‐collection. One month after inoculation with PVY SON41p‐119N, 48.0% of these 254 pepper accessions showed systemic necrosis, while 30.8% showed symptoms of systemic mosaic and 21.2% showed no symptoms at all. No symptoms were observed in mock‐inoculated plants. Globally, results obtained with plant height (Figure 1) and weight (Figure S1) were highly similar. Plant height relative to mock‐inoculated controls ranged from 0.04 to 2.67 (median 0.62; Figure 1a) and 17 accessions had relative plant height greater than 1. Similarly, the relative plant weight ranged from 0.014 to 2.17 (median 0.45; Figure S1a) and 18 accessions had a relative plant weight greater than 1. Both relative plant height and weight were highly variable and showed significant differences between accessions (*p* < 0.001, Kruskal–Wallis test). They also showed high broad‐sense heritability, with *h* ^2^ = 0.97 for relative height and *h* ^2^ = 0.91 for relative weight. A strong correlation (Spearman *ρ* = 0.93, *p* < 0.001) was observed between the relative plant height and relative plant weight. There was also a clear link between the relative plant height (or weight) and the type of symptoms (Figure 1b and Figure S1b) but no link with viral load (Spearman *ρ* = 0.006 and −0.015, *p* = 0.93 and 0.82, for relative plant height and weight, respectively). Previously, the population structure of the pepper core‐collection has been inferred and four genetic clusters had been identified (Tamisier et al., 2020). Among them, three were composed of the cultivated subspecies *C. annuum* var. *annuum* (clusters 1, 2, and 3), while cluster 4 consisted only of the wild subspecies *C. annuum* var. *glabriusculum*. The accession clustering was consistent with the morphological and developmental traits of plants such as fruit shape and length, number of leaves, or date of flowering. For example, the pepper fruits were mostly large in the first cluster, triangular and/or elongated in the second cluster, small and elongated in the third cluster, and tiny and ovoid in the fourth cluster. The relative height of the plants was significantly lower for plants belonging to cluster 2 than for plants belonging to other clusters (Figure 1c). Likewise, the relative weight of the plants was significantly smaller for the plants belonging to cluster 2 than for the plants belonging to clusters 1 and 4 (Figure S1c).

For each plant accession, the slope of the line linking inoculated and mock‐inoculated plants in the plane representing the plant height (or weight) and the within‐plant viral load was used to estimate the tolerance of the plant to PVY infection (Figure S2). Within‐plant viral loads were analysed in a previous study (Tamisier et al., 2020) and overall viral loads as well as the relative plant height and weight could be estimated for 249 of the 254 accessions. Among the 249 pepper accessions, 73 were either virus‐free or had a very low viral load (relative virus accumulation less than 0.10). For these accessions, the tolerance estimation was either meaningless, in cases where the virus was absent, or unreliable because the dots corresponding to the inoculated and mock‐inoculated plants were very close to one another. Therefore, reliable tolerance estimations were obtained for the remaining 176 accessions only. Among pepper accessions, tolerance measured with relative plant height (“height tolerance”) ranged from −270 to 4.70 (median −15.21), with nine accessions having a height tolerance greater than 0. Tolerance measured with relative plant weight (“weight tolerance”) ranged from −119 to 3.38 (median −7.82), with eight accessions showing a weight tolerance greater than 0. The Yeo–Johnson transformation was applied to both tolerance measurements to approximate a normal distribution and to perform the genome‐wide association study (GWAS) (Figure 1d and Figure S1d). A strong correlation (Spearman *ρ* = 0.93, *p* < 0.001) was observed between height tolerance and weight tolerance. Among pepper accessions, both tolerance measurements were lowest for accessions in clusters 2 and 3 and highest for the wild accessions in cluster 4 (Figure 1e and Figure S1e).

### 2.2 Genome‐wide association mapping of pepper tolerance to PVY

**FIGURE 2:**
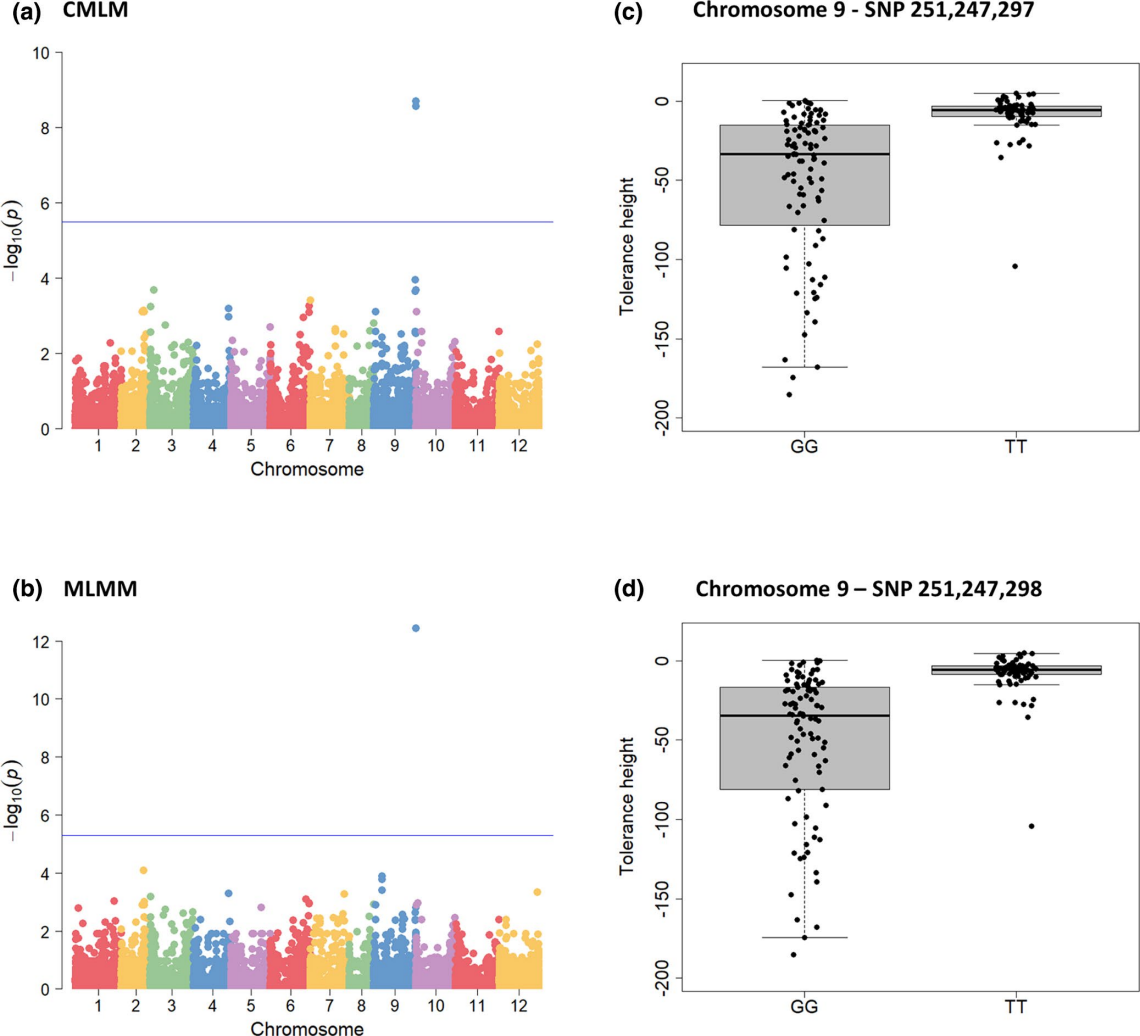
Genome‐wide association studies (GWAS) of potato virus Y height tolerance among the pepper core‐collection. (a, b) Manhattan plot of the compressed mixed linear GWAS model (CMLM) and of the multilocus mixed GWAS model (MLMM), respectively. Negative log_10_(*p*) from genome‐wide scans are plotted against single‐nucleotide polymorphism (SNP) positions on each of the 12 chromosomes. (c, d) Allelic effects at the two significant SNPs, whose positions on the CM334 pepper reference genome are indicated in bp, detected with CMLM and MLMM GWAS models

Genome‐wide association was performed on both height and weight tolerance using the compressed mixed linear model (CMLM) and the multilocus mixed model (MLMM). Two single nucleotide polymorphisms (SNPs) located on chromosome 9 were significantly associated with both tolerance traits (false discovery rate [FDR] and Bonferroni‐corrected threshold <0.05 for CMLM and MLMM, respectively) (Figure 2a,b and Figure S3a,b; Table 1). The first SNP was located at nucleotide position 251,247,297 bp and was detected with both models. The second SNP was adjacent to the first one, at nucleotide position 251,247,298 bp, and was only detected with CMLM. Allelic effects were estimated for the two SNPs and the two tolerance measures (Figure 2c,d and Figure S3c,d). Depending on the model and the tolerance measure, these SNPs explained from 18% to 24% of the phenotypic variation (Table 1).

**TABLE 1:** Single‐nucleotide polymorphisms (SNPs) identified with genome‐wide association studies and associated with height and weight tolerance

Trait	Chromosome number	SNP position (bp)	CMLM		MLMM	
			−log_10_(*p*)	*R* ^2^ (%)	−log_10_(*p*)	*R* ^2^ (%)
Height tolerance	9	251,247,297	8.70	18.86	12.44	24.13
	9	251,247,298	8.57	18.53	NS	NS
Weight tolerance	9	251,247,297	8.18	18.19	10.93	18.21
	9	251,247,298	8.06	17.88	NS	NS

CMLM, compressed mixed linear model; MLMM, multilocus mixed model; NS, nonsignificant.

### 2.3 Candidate genes for PVY tolerance in the pepper genome

The two SNPs (positions 251,247,297 and 251,247,298 bp) associated with tolerance are located within the nucleotide‐binding and leucine‐rich repeat (LRR) domain receptor (NLR) gene CA09g17710, which shares 87.1% nucleotide identity with either the *Bs2* gene from *Capsicum chacoense* (GenBank accession AF202179) that confers resistance to bacterial spot, caused by *Xanthomonas campestris* pv. *vesicatoria* (Tai et al., 1999), or its homolog in *C. annuum* ‘Early Cal Wonder‐20R’ (GenBank accession AY702979). Using the reference genome sequence of *C. annuum* accession CM334, version 1.55 (Kim et al., 2014), a confidence interval containing the candidate genes for tolerance was defined using the closest SNPs that were not detected in GWAS but had major/minor allele frequency ratios similar to those of the significant SNPs (Tamisier et al., 2020) (Table S1). A total of 61 candidate genes were obtained, including 17 resistance gene analogues (RGAs). These RGAs belong to three classes: three of them encode receptor‐like proteins and 13 encode proteins displaying sequence similarity with NLR proteins (Table S1). The confidence interval also includes one gene belonging to the *eIFiso4G* (eukaryotic initiation factor 4G isoform) class of viral recessive resistance genes; this class includes the rice *Rymv1* gene, which confers resistance to rice yellow mottle virus (genus *Sobemovirus*) (Albar et al., 2006). Twenty additional candidate genes have been annotated as being involved in hormone signalling (ethylene or cytokinin), reactive oxygen species (ROS) generation, or as a possible effector target (BED domain‐containing protein) and may also be involved in plant resistance to pathogens.

### 2.4 Mapping of a PVY tolerance gene in biparental progenies and linkage with nematode resistance genes *Me1* and *Me3*

In addition to the core‐collection, three doubled‐haploid (DH) populations were used in this study. They were obtained from the F_1_ hybrids between two inbred lines: Yolo Wonder (YW) and Perennial, PM217, or PM687. Following inoculation with PVY SON41p‐119N, the plants were all scored for symptoms. The parental line YW showed mosaic symptoms at the systemic level while the parental lines Perennial, PM217, and PM687 showed necrotic symptoms. The F_1_ hybrid between Perennial and YW showed systemic necrotic symptoms, suggesting that the necrosis trait is dominant; no PM687 × YW or PM217 × YW F_1_ hybrid plant was available. In the three DH pepper progenies, a clear segregation was observed between DH lines showing necrotic or mosaic symptoms at the systemic level after PVY inoculation (Table 2). For each progeny, the results were consistent with a 1:1 segregation corresponding to a monogenic determinism, even if the probability value was close to the 0.05 threshold for the PM687 × YW DH progeny. For the Perennial × YW DH progeny, comparison with markers covering the pepper genome (Quenouille et al., 2014) revealed that the gene determining necrosis/mosaic segregation was located between the ASC015 and SSCP_MP5 markers on a distal part of chromosome 9 and was closely linked to marker SSCP_MP5, at a distance of 0.96 ± 1.85 Kosambi centimorgans (in short cM in the following) (Lorieux, 1993). This marker was localized between the physical positions 248,725,474 and 248,725,250 bp in the reference pepper genome. In addition, the marker SNP18256 (Quenouille et al., 2014), which is located within the *eIFiso4G* gene mentioned above (Table S1) was estimated to be 8.3 cM distant from the gene responsible for the necrosis/mosaic polymorphism.

**TABLE 2:** Segregations for systemic symptoms after potato virus Y infection observed in three doubled‐haploid (DH) pepper progenies and distance from closest marker or from nematode resistance genes

DH population	Number of DH lines			*p* value^a^ (1:1 segregation)	Name of closest marker or gene	Distance to closest marker or gene (± *SD*)^b^
	Necrotic	Mosaic	Total			
Perennial × YW	107	102	209	0.729	SSCP_MP5	0.96 ± 1.85
PM687 × YW	33	19	52	0.052	*Me3*	3.85 ± 2.68
PM217 × YW	9	8	17	0.808	*Me1*	11.99 ± 8.27

^a^ Chi‐square test.

^b^ Kosambi centimorgans.

In another pepper progeny, the SSCP_MP5 marker was shown to be 8.8 cM distant from the nematode resistance gene *Me1* (marker named SSCP_PM5 in Fazari et al., 2012). A QTL determining the kinetics of symptom expression (area under the disease progress curve, AUDPC) after PVY inoculation was also mapped in the vicinity of SSCP_MP5 in the Perennial × YW DH progeny (Quenouille et al., 2014).

For the PM687 × YW and PM217 × YW DH progenies, necrosis/mosaic segregations were compared to those of nematode (*Meloidogyne incognita*) resistance genes *Me3* and *Me1*, respectively, both located on pepper chromosome 9 (Djian‐Caporalino et al., 2007; Fazari et al., 2012). The gene determining necrotic or mosaic symptoms on PVY infection was shown to be located at distances of 3.85 ± 2.68 cM and 11.99 ± 8.27 cM from *Me3* and *Me1*, respectively (Table 2). In summary, using three independent biparental progenies, a gene determining necrotic or mosaic symptoms after PVY inoculation was shown to be located on pepper chromosome 9, not far from the nematode resistance genes or a linked marker.

### 2.5 Joint evolution of pepper resistance and tolerance to PVY

**FIGURE 3:**
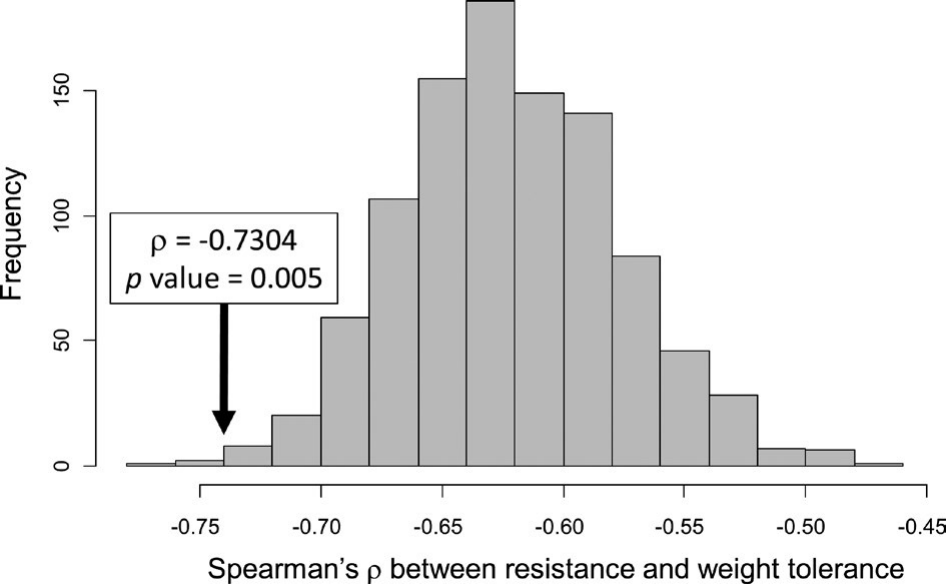
Distribution of Spearman's *ρ* values between resistance and weight tolerance obtained using 1000 random permutations. The *ρ* value between resistance and weight tolerance among pepper accessions and its associated *p* value are indicated

Due to the dependence of the variables used to measure resistance and tolerance, we could not directly analyse the correlation between these two traits among the accessions of the pepper core‐collection. Indeed, resistance was estimated as the opposite (or the reverse) of viral load (i.e., as (−VL) or (1/VL)) and weight tolerance as (Wi − Wm)/VL (see the Experimental Procedures section), VL being the measure of viral load. As a result, VL appears in both variables, leading to a spurious negative correlation (Jackson & Somers, 1991; Strauss & Agrawal, 1999). Stated differently, even if the VL and (Wi − Wm) values are associated randomly among pepper accessions, strong and negative coefficients of correlation (Spearman's *ρ*) between resistance and tolerance estimates are obtained. These *ρ* values deviate strongly from the null hypothesis H_0_: *ρ* = 0 (*p* < 0.001). To circumvent this pitfall, we used two different approaches. First, as suggested by Jackson and Somers (1991), we compared resistance and tolerance traits among accessions by defining an ad hoc null hypothesis based on the distribution of *ρ* values obtained by using random permutations. Among pepper accessions, the Spearman's coefficient of correlation between (Wi − Wm)/VL and (−VL) is *ρ* = −0.730 (Figure 3). The *ρ* values obtained with 1000 random permutations of VL values among pepper accessions ranged from −0.762 to −0.464 (mean −0.623). Only five of those 1000 random permutations produced *ρ* values lower than the *ρ* value calculated on the actual resistance and tolerance data. Therefore, the correlation between resistance and weight tolerance data among pepper accessions is significantly more negative than expected under the null hypothesis defined by random permutations, with a *p* value of 0.005 (Figure 3). Similarly, the correlation between resistance and height tolerance data is significantly more negative than expected under the null hypothesis defined by random permutations, with a *p* value of 0.0006. The correlation between tolerance and resistance has also been analysed among two groups of accessions: the 82 accessions exhibiting leaf necrosis (the least tolerant group) and the 73 accessions exhibiting leaf mosaics (the most tolerant group). In the first group, the correlation between height tolerance and resistance was slightly more negative than expected under the null hypothesis defined by random permutations, with a *p* value of 0.047. In contrast, the correlation between weight tolerance and resistance did not differ from the expectations under the null hypothesis (*p* = 0.116). In the “mosaic” group, the negative correlation was not significantly different from the expectations, for both the height and weight tolerance (*p* = 0.884 and 0.901, respectively).

**FIGURE 4:**
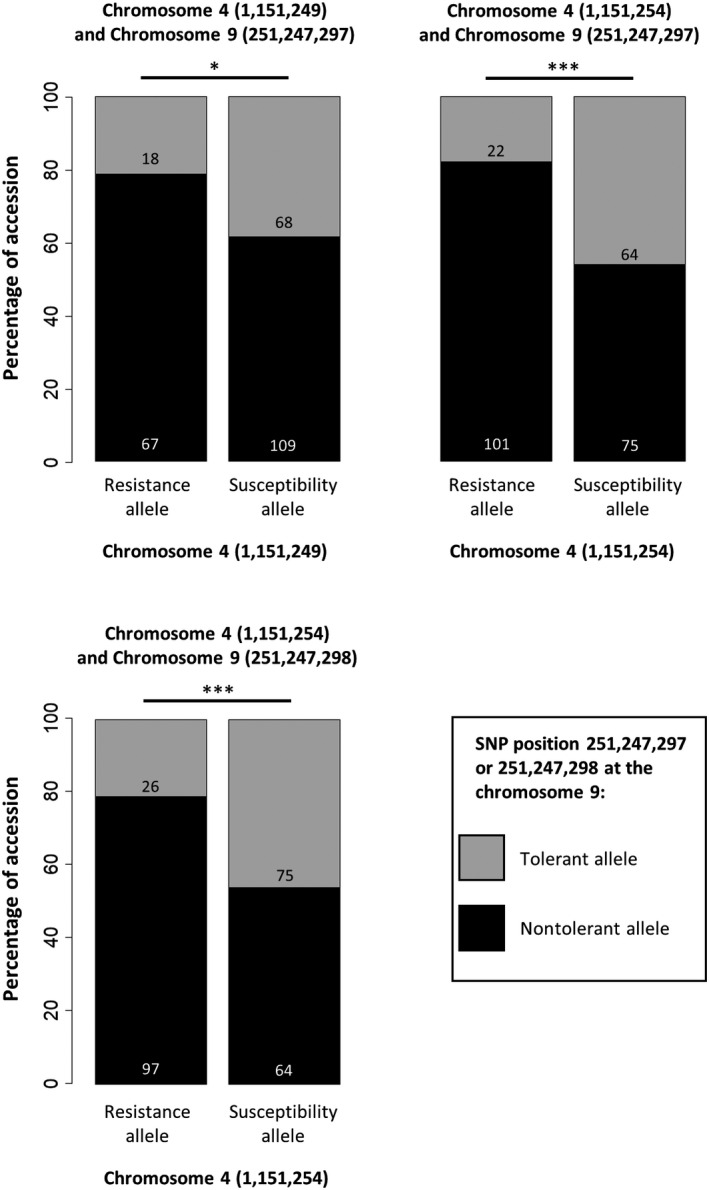
Distribution of the pepper accessions according to the allele they carry for pairs of tolerance and resistance‐associated single‐nucleotide polymorphisms (SNPs). Only the pairs of SNPs showing significant departure from random (after Bonferroni correction for multiple tests) are shown (Table S2). For each pair of SNPs, the two bars split the accessions according to the allele they carry at the resistance‐associated SNP, and the black and grey colours split the accessions according to the allele they carry at the tolerance‐associated SNP. Only homozygous accessions have been used. Fisher exact tests were used to compare accession distribution among the different categories (*p < 0.05 and ***p < 0.001 after Bonferroni correction)

Second, we used a genetics approach to analyse the associations between favourable resistance and tolerance alleles among pepper accessions. A previous GWAS performed on the pepper core‐collection identified seven SNPs linked to pepper resistance to PVY, using CMLM and MLMM (Tamisier et al., 2020). These SNPs were associated with the number of primary infection foci in the inoculated leaves (effective population size of PVY at the inoculation step) and/or the viral load at the systemic level and were localized on chromosomes 4 (positions 1,151,249, 1,151,254, and 340,333 bp), 6 (positions 234,142,995 and 234,143,013 bp), 9 (position 58,056,303 bp), and 12 (position 235,513,719 bp). The SNP on chromosome 9 associated with the effective population size of PVY at the inoculation step is at the other chromosome end compared to the SNPs associated with PVY tolerance identified in the present study. To study the evolution of pepper resistance and tolerance to PVY, the distribution of resistance and tolerance‐associated alleles was analysed for pairs of SNPs, using the 262–266 (depending on the SNP pair) homozygous accessions available (Figure 4 and Table S2).

The favourable resistance and tolerance alleles of the SNPs on chromosomes 4 (positions 1,151,249 and 1,151,254 bp) and 9 (positions 251,247,297 and 251,247,298 bp) were carried together less often than expected by chance (χ^2^ tests, *p* = 8.0e−07 to 0.015, depending on the pair of SNPs). The distribution of all the other pairs of resistance and tolerance‐associated SNPs among the pepper accessions was not significantly different from what might be expected by chance after Bonferroni correction (Figure 4 and Table S2). Importantly, in the pepper collection, there was a clear imbalance between resistance and susceptibility alleles at position 340,333 bp of chromosome 4 (for homozygous accessions, resistance/susceptibility allelic distribution was 22 vs. 241) and on chromosomes 6 (30 vs. 234 for position 234,143,013 bp and 17 vs. 247 for position 234,142,995 bp), 9 (15 vs. 250), and especially 12 (4 vs. 262), which provides little statistical power to analyse their association with the alleles of the SNPs on chromosome 9 linked to tolerance. The distribution of resistance and tolerance‐associated SNP alleles was also different between pepper genetic clusters. The 11 wild accessions of cluster 4 (*C. annuum* var. *glabriusculum*) combined a majority of SNP alleles associated with resistance and tolerance (with the exception of the SNP associated with resistance on chromosome 12, which is very rare in the whole core‐collection). In the three other clusters of cultivated accessions, the resistance and tolerance SNP alleles are in the minority or evenly distributed. Comparison of the co‐segregation of resistance and tolerance‐associated alleles between cultivated (clusters 1–3) and wild (cluster 4) accessions revealed significant deviations for resistance SNPs at positions 1,151,249 and 1,151,254 bp on chromosome 4 when compared to the tolerance SNP at position 251,247,298 bp of chromosome 9 (Table S3). Association between these favourable resistance and tolerance SNP alleles was significantly more frequent among wild than among cultivated accessions. Other co‐segregations of resistance and tolerance‐associated alleles did not differ significantly between cultivated and wild accessions after Bonferroni correction but a trend similar to that above was observed for association of the other resistance SNPs with the tolerance SNP at position 251,247,298 bp of chromosome 9. However, these analyses could suffer from a lack of statistical power because of the small number of wild accessions.

## 3 DISCUSSION

Many studies have focused on analysing plant resistance to parasites, providing us with an in‐depth understanding of its molecular and physiological mechanisms and how it evolves. Much less data is available on tolerance, the second important plant defence strategy against parasites. Moreover, in many cases the effect of the parasite on plant health is measured by scoring disease symptoms, which does not allow the respective roles of resistance and tolerance to be disentangled because a reduction of the disease level can be caused by one of these mechanisms or a combination of both. The simultaneous measurement of the within‐plant viral load and the impact of the viral infection on plant growth allowed us to estimate PVY tolerance quantitatively on a pepper core‐collection using the slope method (Figure S2) (Råberg et al., 2007). We have shown that tolerance is largely determined by a single locus located on chromosome 9 in a genomic region rich in resistance gene analogues. This is in agreement with the observation that only a few QTLs of major effect generally control virus tolerance (Pagán & García‐Arenal, 2020).

Tolerant accessions generally exhibited no symptoms or leaf mosaics at the systemic level while nontolerant accessions exhibited leaf necrosis or a mixture of necrosis and mosaics. Necrosis started along the veins and spread throughout the whole leaf blade, petiole, and plant stem. These symptoms are reminiscent of those induced by necrotic strains of PVY in tobacco (Michel et al., 2018). In three pepper biparental progenies, a locus in the same region of chromosome 9 has been shown to determine the occurrence of systemic necrosis or mosaic disease during PVY infection. Phenotypically, the necrotic symptoms seen in nontolerant pepper accessions resemble hypersensitive reactions insufficient to confine the virus in the inoculated leaves. Several cases of systemic necrosis triggered by viral infection have been shown to be caused by resistance genes or resistance gene analogues, such as the *Rx* and *NtTPN1* genes (Baurès et al., 2008; Michel et al., 2018). In particular, the *NtTPN1* gene has been shown to be involved in the tolerance of tobacco plants to a necrotic strain of PVY (Michel et al., 2018). Many resistances to pathogens conferred by NLR proteins are inhibited at high temperatures (28°C or above). Pepper systemic necrosis induced by PVY is also temperature sensitive. Almost no necrosis is visible when the plants are kept constantly at 28°C, while at a low temperature (constant 20°C) the necrosis frequently evolves towards plant death about 1 month after inoculation (data not shown). The present study was carried out under greenhouse conditions where temperatures varied from 22 to 25°C and promoted the expression of necrosis that rarely led to plant death.

Consistent with the hypothesis that a mechanism similar to hypersensitivity might be involved in PVY lack of tolerance, the genomic interval of candidate genes around the detected SNPs contained a large set of NLR genes, including analogues of *Bs2*, which are the most obvious tolerance gene candidates (Table S1). The nematode resistance genes *Me1* and *Me3* were also located in this part of chromosome 9 and are 3.85–11.99 cM distant from the gene responsible for necrosis during PVY infection (Table 2). A gene encoding an eIFiso4G is located at a boundary of the candidate gene interval. This gene seems unlikely to be involved in the PVY tolerance trait, as eIF‐mediated viral resistance is generally recessive (the PVY necrosis trait is dominant, as shown by the Perennial × YW F_1_ hybrid) and is not associated with cell death.

An important pending question is the identification of the factors that determine the evolution of resistance or tolerance in plants, notably the existence of genetic trade‐offs between these two defence traits (reviewed in Pagán & García‐Arenal, 2018, 2020; Strauss & Agrawal, 1999). No global trade‐off was found among all the pepper accessions. However, both trait‐based and genetic‐based analyses have revealed a negative correlation between resistance and tolerance within the cultivated accessions clusters. This correlation was more negative than expected under an ad hoc null hypothesis based on random permutations. Two SNPs associated with PVY tolerance were mapped using the pepper core‐collection. Seven SNPs, corresponding to five loci, were previously associated with PVY resistance (Tamisier et al., 2020). While the tolerance SNPs are located at one end of chromosome 9, the resistance loci are either located on other chromosomes (4, 6, and 12) or at the other end of chromosome 9. Among the pepper collection, resistance alleles at different loci tended to be associated more frequently than expected by chance, which could be explained by a higher efficiency or durability of resistance when multiple resistance alleles are combined (pyramiding of resistance QTLs) (Tamisier et al., 2020). Conversely, the analysis of associations of SNP alleles linked to resistance and tolerance (Figure 4) revealed a negative correlation between these two defence traits, which varied between the pepper genetic clusters. In particular, the wild accessions of cluster 4 combined SNP alleles associated with resistance and tolerance whereas resistance and tolerance SNP alleles were not predominant among cultivated accessions and tended to exclude each other. This suggests that wild pepper accessions have developed a combination of resistance and tolerance traits to PVY, while domestication or farmers' selection have shifted defence mechanisms either toward resistance or tolerance, but rarely toward their combination.

Negative correlations between resistance and tolerance have been shown in several plant–insect pathosystems. For example, Agrawal and Fishbein (2008) have shown an increase in tolerance and a decrease in resistance to herbivorous insects during the evolution of milkweed (*Asclepias* spp.). However, evidence for trade‐offs based on genetic correlations is scarce (Fineblum & Rausher, 1995; Stowe, 1998) and unsystematic (Mauricio et al., 1997). In the case of plant pathogens, the few studies that have examined plant resistance and tolerance simultaneously have led to disparate results (Baurès et al., 2008; Goss & Bergelson, 2006; Kover & Schaal, 2002). More recently, Mikaberidze and McDonald (2020) have shown a trade‐off between tolerance and resistance of wheat to *Zymoseptoria tritici*. Interestingly, after splitting their cultivars into two groups more or less tolerant than the baseline, the negative correlation between tolerance and resistance was only detected in the intolerant group. The same trend was observed for the negative correlation between height tolerance and resistance in our study, the negative correlation being present among the “necrotic” accession group (mainly composed of nontolerant accessions) while it was absent among the “mosaic” accession group (mainly composed of tolerant accessions). However, the trade‐off between tolerance and resistance of wheat to *Z. tritici* was only based on phenotypic correlation and the underlying genetic basis has not been studied yet.

Two hypotheses could explain the negative correlation found between resistance and tolerance among the cultivated accessions. First, if resistance or tolerance alone were enough to defend crops against PVY, domestication or farmers' selection could have selected for either resistance or tolerance, but not both. The negative correlation observed would not be the result of a trade‐off (i.e., the plant inability to simultaneously maximize resistance and tolerance), but would only be due to farmers' selection. Second, a real trade‐off between resistance and tolerance could have evolved among the cultivated accessions. There have been numerous attempts to model the evolution of host resistance and tolerance. It has been suggested that resource availability, plant growth rate, lifespan, and the costs and benefits associated with each defence strategy condition the evolution of plants towards resistance, tolerance, or both (discussed in Pagán & García‐Arenal, 2018). As resource availability is generally higher for crops than for wild plants, it seems easier for cultivars than for their wild relatives to accommodate both resistance and tolerance mechanisms. However, the allocation of resources also contrasts drastically between crops and wild plants. Crop plants were selected to allocate much more resources towards biomass and specific organs (leaves, fruit flesh) which may have no direct link with plant fitness. Therefore, their resource allocation strategies are expected to be significantly different from those of wild plants. The costs of tolerance and resistance mechanisms may thus be higher for cultivated accessions than for wild accessions, explaining the fact that a resistance–tolerance trade‐off would only be observed among the cultivated pepper accessions.

Finally, from an applied perspective, it has already been shown in the pepper–PVY and other pathosystems that combining a major‐effect resistance gene with resistance QTLs increases resistance durability. The use of the tolerance locus identified here would be another strategy to control PVY‐induced disease in pepper. In theory, tolerance is expected to exert little (if any) selection on the pathogen populations and is therefore expected to be durable. Moreover, as the PVY tolerance trait is controlled by a single locus, it would be relatively simple for breeders to implement a screening scheme to select pepper varieties carrying the tolerance allele.

## 4 EXPERIMENTAL PROCEDURES

### 4.1 Plant and virus material

Four different populations of *C. annuum* were used. The first one is a core‐collection of 310 accessions, defined to maximize the allelic diversity among a set of simple‐sequence repeats (SSR) markers that was previously used to map PVY resistance QTLs by GWAS (Tamisier et al., 2020). For GWAS, 10,308 SNPs covering the entire pepper genome were obtained by double‐digest restriction associated DNA sequencing (ddRADseq) for 276 of the 310 accessions and PVY resistance was measured for 256 of the 310 accessions (Tamisier et al., 2020).

In addition, three doubled‐haploid (DH) populations were obtained from the F_1_ hybrids between two inbred lines: on one side Yolo Wonder (YW) and on the other side Perennial, PM217 (also named PI201234), or PM687 (also named PI322719). Yolo Wonder is susceptible to PVY isolates. Perennial carries the PVY resistance allele *pvr2^3^* and several PVY resistance QTLs (Caranta et al., 1997; Quenouille et al., 2014). PM217 and PM687 are resistant to the nematode species *M. incognita*, a trait conferred by the *Me1* and *Me3* genes, respectively (Fazari et al., 2012). A genetic map of the Perennial × YW DH progeny was obtained previously (Quenouille et al., 2014).

To measure pepper tolerance, we used the PVY clone SON41p‐119N derived from isolate SON41p. This clone carries the Asp_119_Asn substitution at amino acid position 119 in VPg, which allows it to overcome the *pvr2^3^* resistance, one of the most common resistance alleles among pepper accessions (Ayme et al., 2006; Quenouille et al., 2016).

### 4.2 Phenotyping of the core‐collection: tolerance evaluation

#### 4.2.1 Plant health and virus load

PVY tolerance was estimated on the same plants that were previously analysed for PVY resistance, corresponding to 256 accessions (Tamisier et al., 2020). Fifteen plants per accession were sown in a greenhouse. The PVY clone was multiplied in *Nicotiana tabacum* ‘Xanthi’ plants. Twenty‐eight days after sowing, the two cotyledons of 10 plants per accession were mechanically inoculated with PVY. The remaining five plants per accession were mock‐inoculated for use as uninfected control plants. The infected and noninfected control plants were sown in the same tray. Twice a week, each tray was moved to another spot in the greenhouse to minimize local environmental effects. Thirty days postinoculation (dpi), symptoms on the apical leaves were recorded and the effect of PVY infection on plant health was assessed by measuring the height and fresh weight of inoculated and control plants. The plants were cut at the cotyledon node and immediately measured and weighted. Only the fresh weight was measured because the fresh and dry weights of the pepper plants were previously found to be highly correlated (*r* = 0.957, *p* < 0.0001) (Montarry et al., 2012). The same plants have been used to perform a quantitative double‐antibody sandwich (DAS)‐ELISA and estimate the virus load relatively to a positive control included on all ELISA plates. These virus loads allowed to map the QTLs of resistance to PVY by GWAS on the same plant core‐collection (Tamisier et al., 2020). For each pepper accession, three viral load measurements were obtained as the 10 inoculated plants were pooled into groups of two or four before the quantitative DAS‐ELISA.

#### 4.2.2 Tolerance estimation

The PVY tolerance level of each pepper accession was assessed using a method derived from the range tolerance approach defined by Råberg et al. (2007). Two measurements of tolerance were obtained per accession, using either plant height or plant fresh weight as a proxy of plant health. First, for each accession, the mean height (or weight) of the control plants was calculated. Next, the mean height (or weight) and the mean viral load of the inoculated plants were also calculated. For each accession, two points were plotted in a graph representing plant health against virus accumulation, one for control plants (with a value of 0 for virus accumulation) and one for inoculated plants. The slope of the line linking the two points represented the tolerance level of the accession (Figure S2). Thus, for each plant accession, the weight tolerance can be expressed as: WT = (Wi − Wm)/VL, where Wi is the mean weight of infected plants, Wm is the mean weight of mock‐inoculated plants and VL is the mean viral load in infected plants and the height tolerance as HT = (Hi − Hm)/VL, where Hi is the mean height of infected plants and Hm is the mean height of mock‐inoculated plants.

### 4.3 Phenotyping of the biparental pepper populations: scoring of systemic symptoms

The three different segregating DH populations described above were mechanically inoculated with PVY SON41p‐119N on both cotyledons, 3 weeks after sowing. For each pepper population, 10 plants per line were inoculated and scored. Symptoms on the apical leaves were followed up to 1 month after inoculation and infections were confirmed by DAS‐ELISA. As symptomatology was inherited as a monogenic Mendelian trait in the three DH populations (see the Results section), we tested its linkage with molecular markers already available (Quenouille et al., 2014) or with nematode resistance genes (Fazari et al., 2012).

### 4.4 Genome‐wide association study

We used two different models to perform the GWAS. The first one was the CMLM (Zhang et al., 2010) implemented in the Genomic Association and Prediction Integrated Tool (GAPIT) R package (Lipka et al., 2012). The CMLM takes into account the population structure, using principal components as fixed effects, and the relatedness between accessions by including a random‐effect kinship matrix (K matrix). The FDR was used to adjust for multiple testing and determine a corrected significance level. The second model was the MLMM, which is based on a stepwise mixed‐model regression with forward inclusion and backward elimination (Segura et al., 2012). At each step of the regression, the genetic and error variances are re‐estimated. The advantages of this latter model are that it allows the use of multiple cofactors and takes into account better the confounding effects of background loci. This model also integrates a correction for the population structure (principal components) and for relatedness (K matrix). Finally, for MLMM, the best model was chosen using the multiple‐Bonferroni criterion (mBonf).

A confidence interval around the detected SNPs was calculated. Usually, it is assessed using the mean linkage disequilibrium (LD) decay measured among the accessions. In our study, the rapid LD decay observed did not allow us to use this method. The confidence interval was therefore calculated as described in Tamisier et al. (2020). Upper and lower boundaries were defined as the closest nondetected SNP showing a similar major/minor allele frequency ratio as the one of the significant SNP. The rationale is that if these SNPs were involved in the phenotypic trait analysed, they should have been identified with GWAS because there is no lack of power because of a greater major/minor allele frequency imbalance.

## Data Availability

The data that support the findings of this study are available from the corresponding author upon reasonable request.
