# Supplementary figures and images for: Concurrent evolution of resistance and tolerance to potato virus Y in Capsicum annuum revealed by genome‐wide association
Source: Mol Plant Pathol. 2021 Nov 2;23(2):254–64. doi: 10.1111/mpp.13157 (PMC8743019; doi:10.1111/mpp.13157)

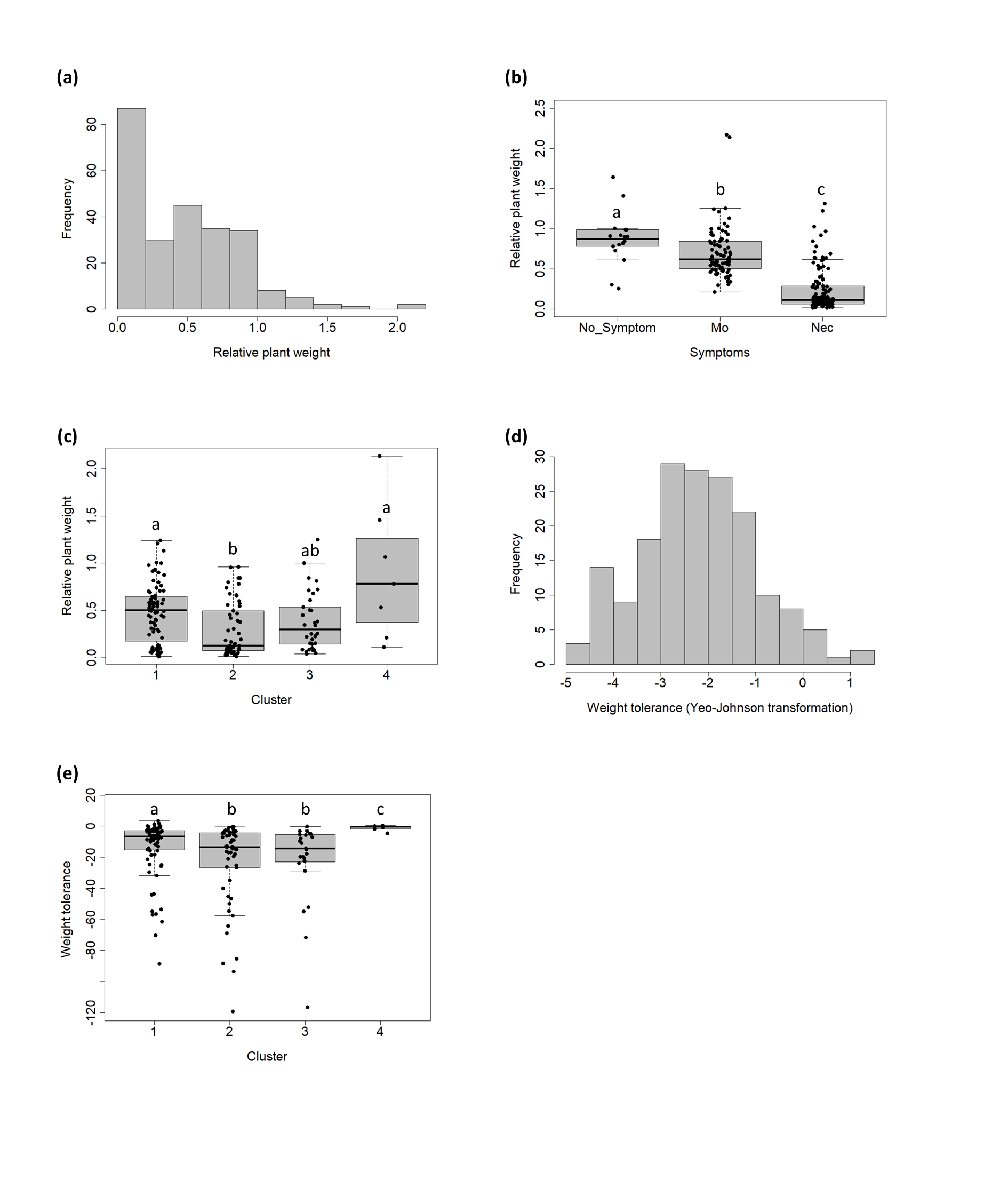

Supplement: Supplementary file 1 [file MPP-23-254-s002.tif]

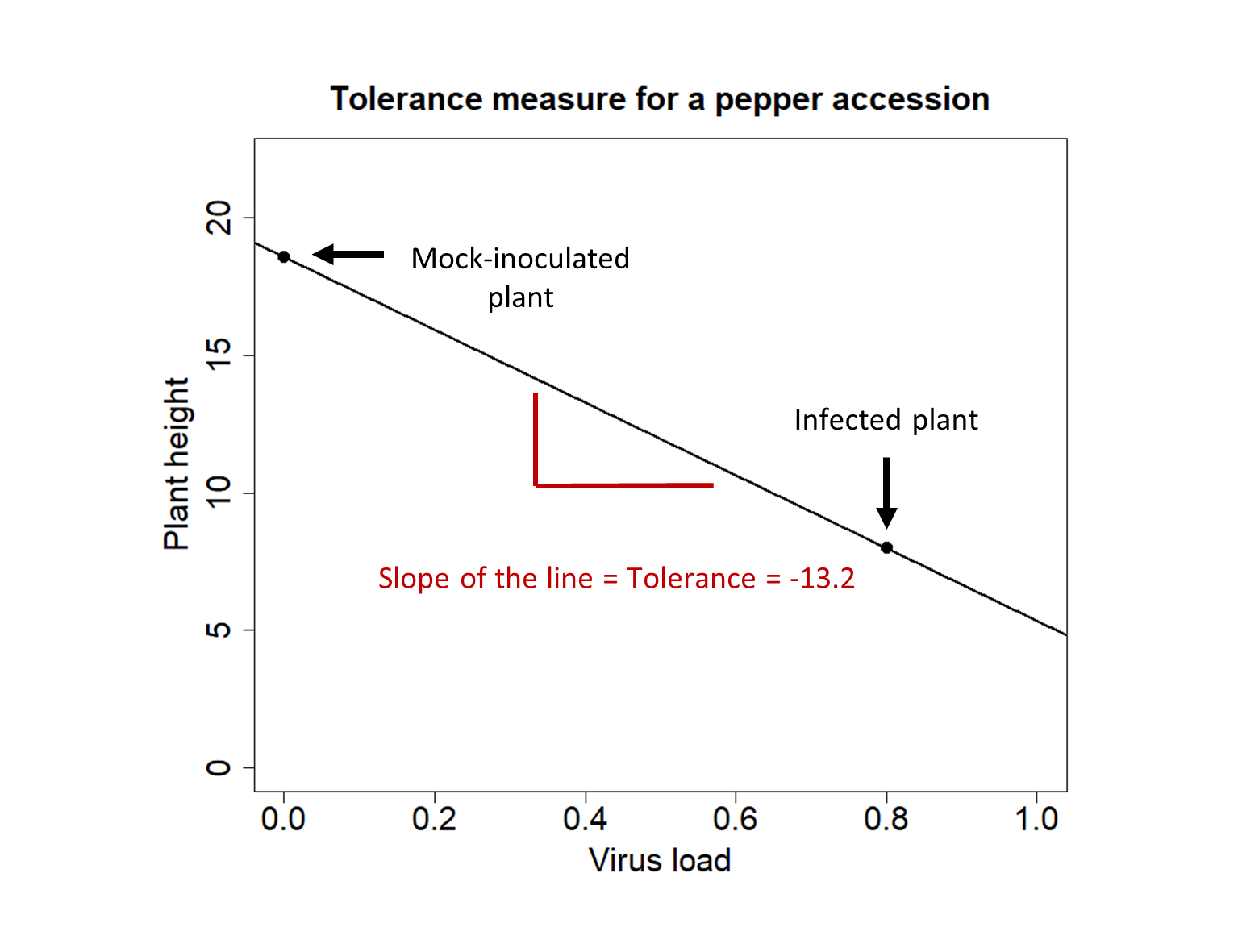

Supplement: Supplementary file 2 [file MPP-23-254-s005.tif]

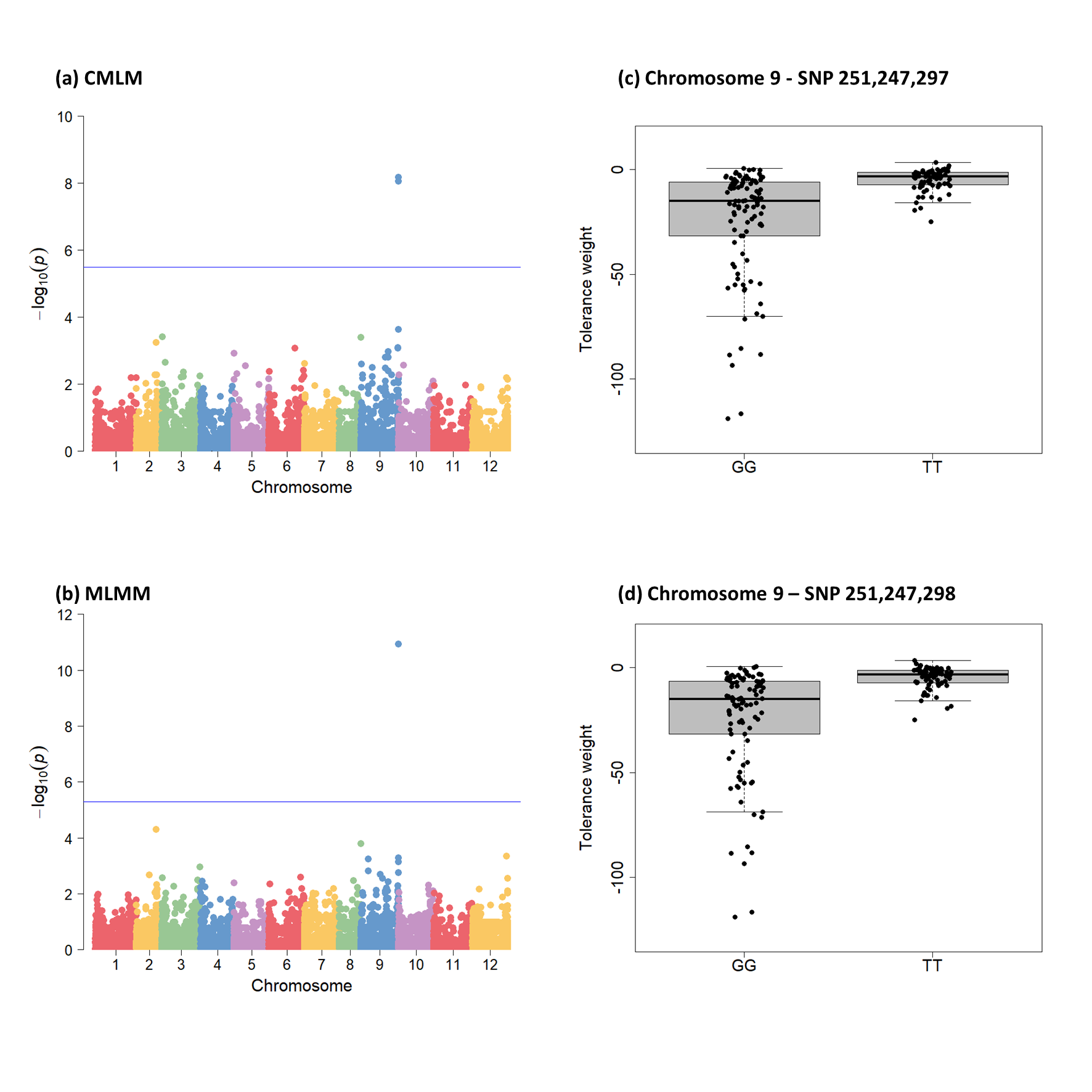

Supplement: Supplementary file 3 [file MPP-23-254-s006.tif]
